# Supplementary material for: Effects of Dietary Supplementation with Dihydromyricetin on Hindgut Microbiota and Metabolite Profiles in Dairy Cows
Source: Microorganisms. 2025 Dec 21;14(1):20. doi: 10.3390/microorganisms14010020 (PMC12843980; doi:10.3390/microorganisms14010020)
Supplement: Supplementary file 1 [file microorganisms-14-00020-s001.zip › Supplementary Figures.pdf]

Supplementary Figures of Manuscript (ID microorganisms-4024708)

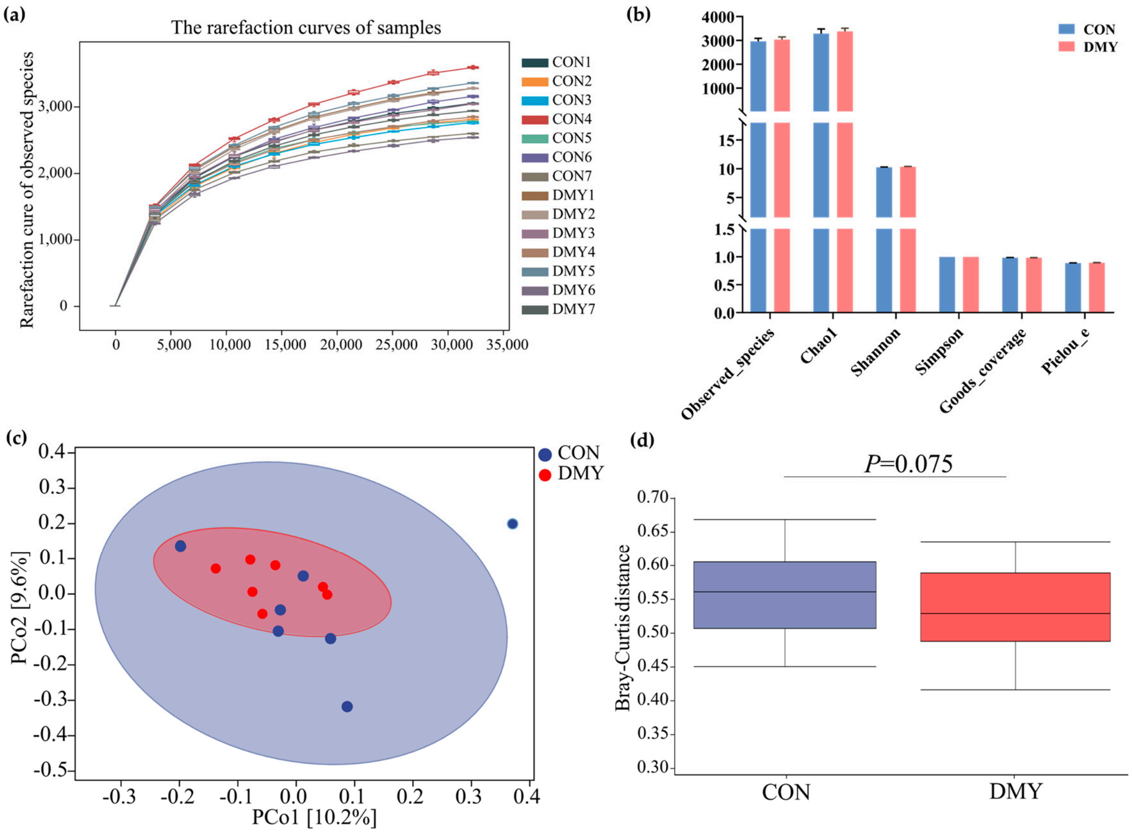

Figure S1. **Variations in gut microbial diversity of dairy cows fed with DMY**. (**a**) The rarefaction curves of observed species for all samples; (**b**) column diagram of α diversity indexes: observed species, Chao1, Shannon, Simpson, Goods coverage and Pielou-e, respectively; (**c**) PCoA plot based on Bray-Curtis metrics; (**d**) Bray-Curtis distance between groups.

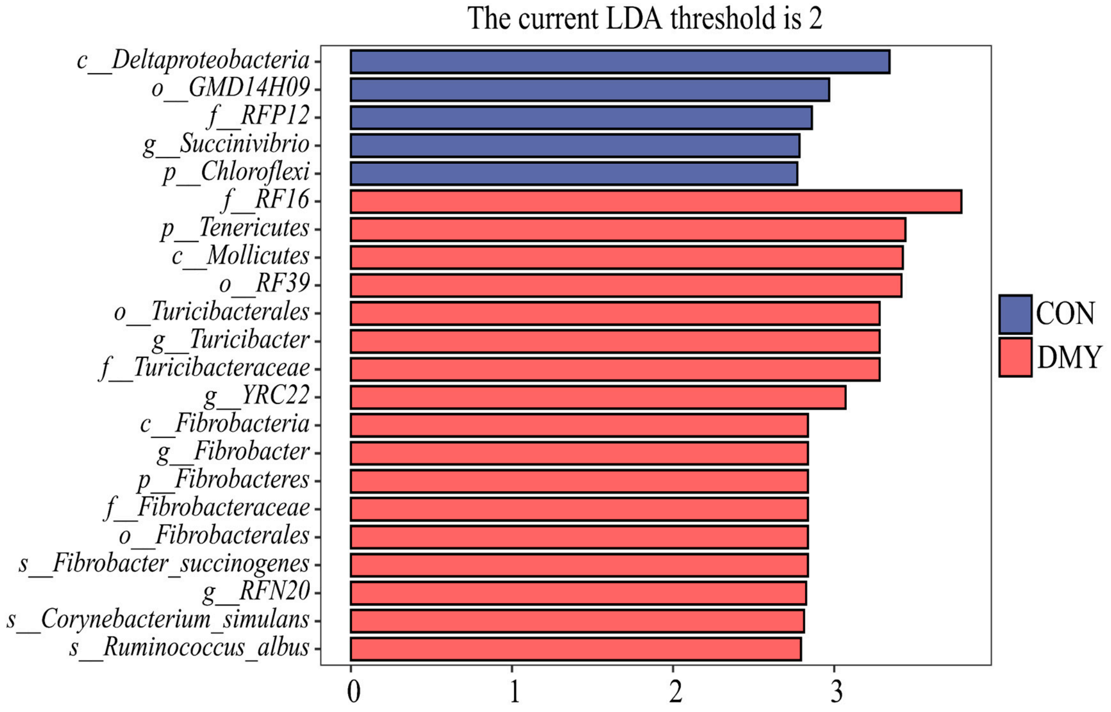

Figure S2. Linear discriminant analysis (LDA) score plot of differential microbial taxa between groups.

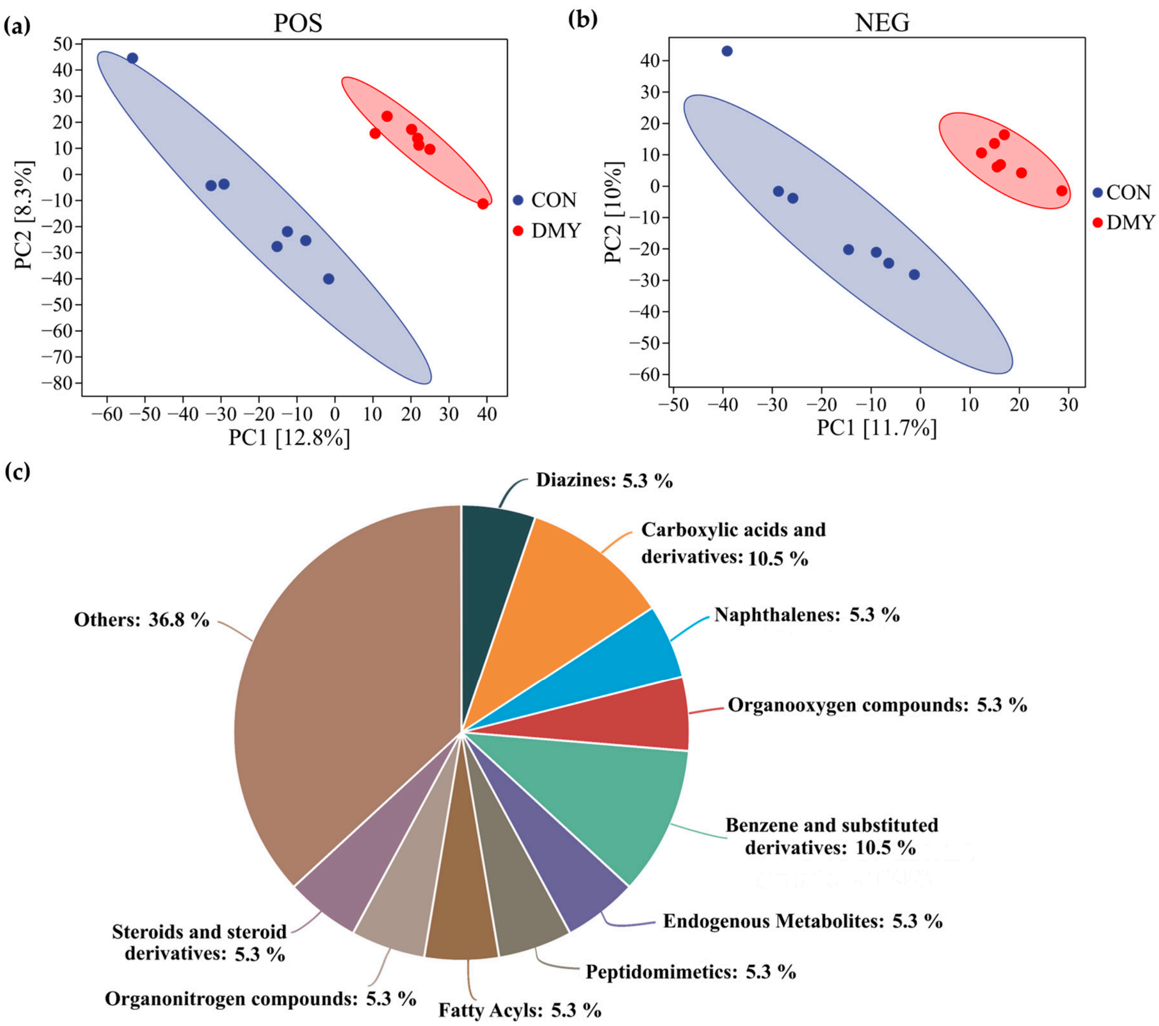

Figure S3. Multivariate statistical PLS-DA analysis and classification of differential metabolites in the fecal metabolome. PLS-DA of detected metabolites under positive ion mode (**a**) and negative ion mode (**b**) in feces; (**c**) the top10 classification of fecal differential metabolites of dairy cows in the CON and DMY groups at subclass level.

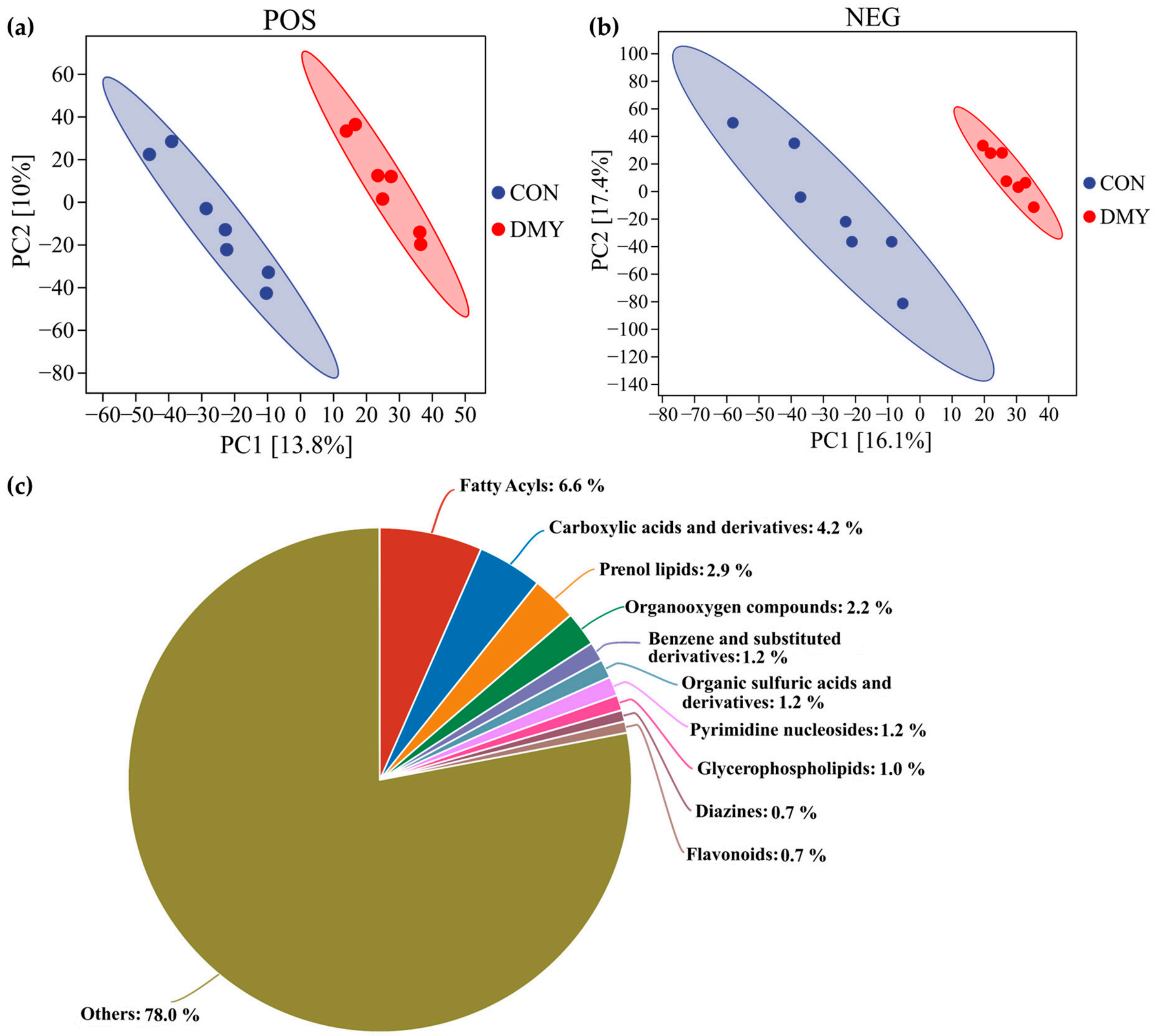

Figure. S4. Multivariate statistical PLS-DA analysis and classification of differential metabolites in the plasma metabolome. PLS-DA of detected metabolites under positive ion mode (**a**) and negative ion mode (**b**) in plasma; (**c**) the top10 classification of plasma differential metabolites of dairy cows in the CON and DMY groups at subclass level.
